# Construction and Validation of a Necroptosis-Related Gene Signature for Predicting Prognosis and Tumor Microenvironment of Pancreatic Cancer

**DOI:** 10.1155/2022/9737587

**Published:** 2022-06-14

**Authors:** Cheng Ding, ZhangPing Yu, JiQiao Zhu, XianLiang Li, MengHua Dai, QiangHe

**Affiliations:** ^1^Department of Hepatobiliary Surgery, Beijing Chao-Yang Hospital, Capital Medical University, Beijing, China; ^2^Department of General Surgery, Peking Union Medical College Hospital, Chinese Academy of Medical Sciences and Peking Union Medical College, Beijing, China

## Abstract

Pancreatic cancer (PC) is notorious for its parallel morbidity and mortality rates. Recently, necroptosis, a form of programmed cell necrosis, has gained popularity for its role in tumorigenesis and metastasis. In this study, we explored the expression of necroptosis-related genes in PC and normal pancreatic tissues and identified 52 differentially expressed genes (DEGs). The Cox regression analysis was applied to construct the prognostic risk model, which divided patients into high- and low-risk groups. PC patients in the low-risk group showed a significantly better overall survival (OS) than those in the high-risk group. We further validated the prognostic role in ICGC cohort. Further, Gene Ontology (GO), Kyoto Encyclopedia of Genes and Genomes (KEGG), Gene Set Enrichment Analysis (GSEA), and tumor microenvironment (TME) analysis were used to explore the underlying mechanisms. Notably, based on the gene signature, we revealed that the risk score was strongly related to the sensitivity of chemotherapy. In conclusion, necroptosis-related genes serve as an important immune mediator, and the risk model could be used to predict the survival and to guide the development of precision drugs for patients with PC.

## 1. Introduction

Pancreatic cancer (PC) is an extremely poor survival digestive system malignancy. PC has high recurrence, metastasis, and mortality rates, which causes 466 000 deaths all over the world in 2020 [1]. Due to insidious symptoms and the difficulty of early diagnosis, 80% of the patients with PC have no chance of surgery at the time of diagnosis [2]. Despite numerous studies being focused on PC, the 5-year overall survival (OS) rate of PC was only 10% [3]. Systemic treatment is the only choice of treatment for PC patients who had lost the chance of surgery. However, the current treatment strategies only marginally improve the survival of PC patients. Moreover, the progress in the treatment of PC is slow when compared to other malignancies. Accordingly, it is imperative to innovate a prognostic model for managing pancreatic cancer.

Necroptosis, a novel programmed form of necrotic cell death, plays a significant role in the host's defense against pathogenic invasion [4]. Necroptosis is morphologically similar to necrosis and mechanistically resembles apoptosis [5]. Apart from its key role in viral infection and inflammatory diseases, it has been demonstrated to show vital effect in tumor biological behavior, tumorigenesis, immunity, invasion, and metastasis [4, 6]. Necroptosis can activate RIPK1 and RIPK3 in the tumor microenvironment (TME) to promote antitumor immunity [7]. Koo et al. reported that the expression of RIPK3 is reduced in breast cancer tissues, and low RIPK3 level indicates poor survival in patients with breast cancer [8]. Similarly, RIPK3 was decreased in colorectal cancer and acute myeloid leukemia and the downregulation of RIPK3 hampered the survival of patients. In contrast, it is reported that RIPK1 and RIPK3 are overexpressed in pancreatic cancer tissues, and downregulation of RIPK1 or deletion of RIPK3 *in vivo* inhibited tumor progression via enhancing immune cell infiltration [9].

Given the interesting reports, we hypothesize that necroptosis might play a dual role in both the progression of tumor and antitumor processes; however, to date, only a few researches have systematically analyzed the effect of necroptosis-related genes in patients with PC. Hence, we conducted an integrative research to evaluate and compare the expression of necroptosis-related genes between PC and normal pancreatic tissues. Moreover, we assessed the correlation between necroptosis and TME in PC, as well as the underlying mechanism, and provide an effective model for prognosis of patients with PC.

## 2. Materials and Methods

### 2.1. Raw Data Gathering

RNA-seq data in TPM format of pancreatic cancer samples from TCGA and normal pancreas tissues from GTEx were obtained from UCSC XENA (https://xenabrowser.net/datapages/), and the RNA-seq data were processed in unison by the Toil process [10]. The RNA-seq data in TPM format and clinical information of the external validation cohort were acquired from the ICGC (https://icgc.org) database. No ethics approval was required as all the raw data was sourced from the public databases.

### 2.2. Selection of Differentially Expressed Necroptosis-Related Genes

The 68 necroptosis-related genes were extracted from the GeneCards database (https://www.genecards.org) with *Z*‐score > 1 (Supplementary Table 1). The R Studio and “limma” package were applied for screening differentially expressed genes (DEGs) with the criteria of FDR < 0.01 and absolute log2FC > 1. The protein-protein interaction (PPI) network of the DEGs was constructed via the Search Tool for the Retrieval of Interacting Genes (STRING) (https://string-db.org). The “igraph” and “reshape2” packages were adopted to construct the correlation network of DEGs.

### 2.3. Unsupervised Clustering of DEGs

To determine the different necroptosis modification patterns and classification of PC patients for further analysis, we conducted unsupervised cluster analysis according to the expression of DEGs. The “ConsensusClusterPlus” package was used for the cluster identification analysis.

### 2.4. Enrichment Analysis

After identifying the differentially expressed necroptosis-related genes between the subtypes categorized by the risk score model, the Gene Ontology (GO) and Kyoto Encyclopedia of Genes and Genomes (KEGG) pathways were used to evaluate the potential biological functions and mechanistic pathways. The above analysis was performed by the “clusterProfiler” package with the criteria of FDR < 0.05 and absolute log2FC > 1. To determine the biological difference between the high- and low-risk groups, we performed the Gene Set Enrichment Analysis (GSEA) via GSEA software. “c2.cp.kegg.v7.4.symbols.gmt” and “h.all.v7.4.symbols.gmt” gene sets were selected as the reference.

### 2.5. Building and Validating the Prognostic Necroptosis-Related Gene Model for PC

After determining the DEGs, firstly, we first applied the univariate Cox regression analysis to identify necroptosis-related genes significantly associated (*p* < 0.05) with overall survival (OS). Then, to narrow down the candidate markers, we performed multivariate Cox regression analysis to identify ultimate necroptosis-related genes and predict the regression coefficients (*β*) of the risk model. Finally, a prognostic risk model according to three genes was constructed. Risk score = (*β*1 × the expression of GSK3B) + (*β*2 × the expression of UCHL1) + (*β*3 × the expression of AIFM1). Based on the median risk score, all PC patients were divided into high- and low-risk groups. To further validate the accuracy and stability of the risk model, the PACA-CA cohort of ICGC database was extracted. We applied the same formula and cutoff value according to the risk model of TCGA cohort. Kaplan-Meier (K-M) survival curves and receiver-operating characteristic (ROC) curves were used to evaluate the predictive values of the risk model. Furthermore, we evaluated the protein level of these three genes in PC via the human protein atlas (HPA) database (https://www.proteinatlas.org).

### 2.6. Independent Prognostic Analysis

To assess whether necroptosis-related risk model is an independent risk factor for the prognosis of PC patients, we performed univariate and multivariate Cox regression analysis combined with age, sex, grade, stage, and risk score in TCGA cohort.

### 2.7. Tumor Immune Microenvironment Analysis

CIBERSORT [11, 12], a deconvolution algorithm based on support vector regression, was applied to calculate the tumor immune infiltration cells in PC patients. We also used the “estimate” package to calculate the ratio of immune-stromal cells in TME. We exhibited the results of ImmuneScore, StromalScore, and ESTIMATEScore with boxplot. Increasing focus on the level of immune checkpoint genes and the chosen of immunotherapy, hence, we further compared the common key immune checkpoint genes, PD1 (PDCD1), PD-L1 (CD274), B7-H3 (CD276), CTLA4, LAG3, and TIGIT included, between the high- and low-risk groups. We adopted boxplots to demonstrate the differences between these two groups.

### 2.8. Associations between Risk Score and Drug Treatment

To evaluate the association between the necroptosis-related gene risk model and drug sensitivity in patients with PC, we adopted pRRophetic and ggplot2 packages. Additionally, we compared the half-maximal inhibitory concentration (IC50) of conventional chemotherapy drugs between the low- and high-risk groups for PC.

## 3. Results

### 3.1. DEG Identification

**Figure 1:**
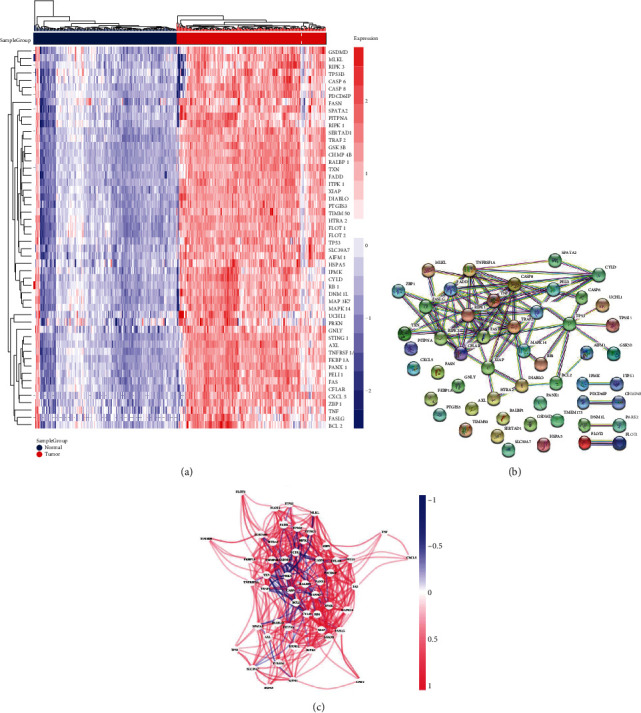
Necroptosis-related gene expressions in PC and their correlations. (a) Heatmap displaying the DEGs between the PC tissues and normal pancreatic samples (blue: low-expression level; red: high-expression level). (b) Protein-protein interaction (PPI) network constructed using STRING database (interaction score = 0.90). (c) The correlation network of the differentially expressed necroptosis-related genes.

The flow chart of this study is displayed in Figure S1. To acquire the DEGs between PC samples and normal pancreatic tissues, we compared the level of 68 necroptosis-related genes in 178 PC samples and 171 normal tissues obtained from TCGA and GTEx databases. 52 DEGs were identified, and all of these genes were overexpressed in PC tissues (Figure 1(a)). To further analyze the interactions between these necroptosis-related DEGs, we constructed the protein-protein interaction (PPI) network by the STRING database with the highest confidence (0.90) (Figure 1(b)). Additionally, we built a correlation network of these DEGs (Figure 1(c)). Furthermore, we applied GO and KEGG enrichment analysis for these DGEs. As shown in Figure S2, the DEGs were enriched in “regulation of apoptotic signaling pathway,” “membrane region,” “ubiquitin-like protein ligase binding,” and “necroptosis.”

### 3.2. Cluster and TME Analysis

**Figure 2:**
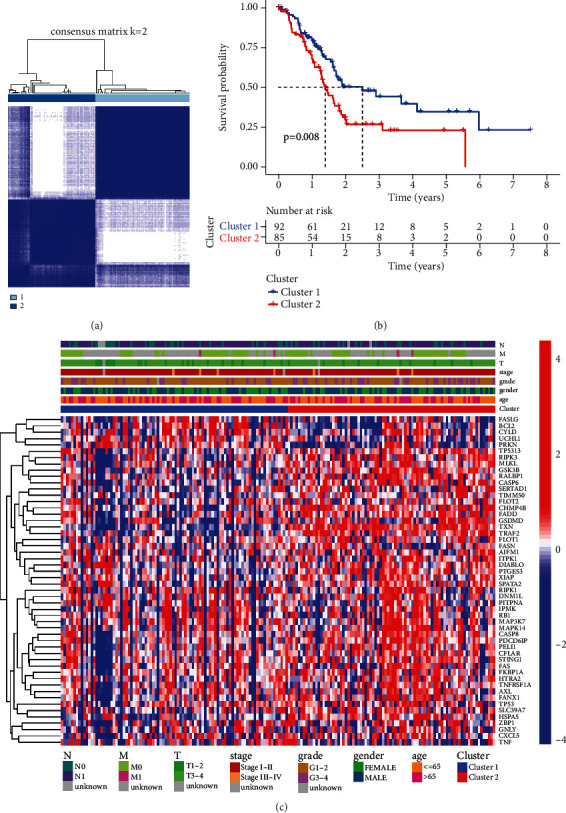
Clusters based on the necroptosis-related DEGs. (a) Two different clusters were identified in patients with PC by unsupervised clustering method. (b) Kaplan-Meier survival analysis showed that the cluster 1 PC patients had a significantly better overall survival (OS) than cluster 2 (*p* = 0.008). (c) Heatmap of necroptosis-related DEGs and clinicopathologic features.

We performed an unsupervised clustering analysis based on the expression of 52 necroptosis-related DEGs in the TCGA datasets. By increasing the clustering variable (*k*) from 2 to 9, we identified that PC patient could be divided into 2 subgroups (Figure 2(a)). K-M survival analysis indicated that PC patients in cluster1 had a better OS than those in cluster (Figure 2(b)). In addition, the association between necroptosis-related genes and clinicopathological features was evaluated (Figure 2(c)).

**Figure 3:**
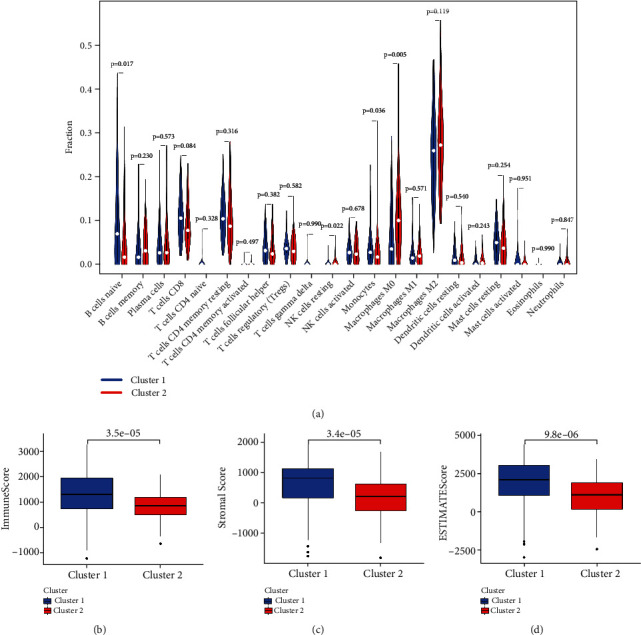
TME analysis based on two clusters. (a) Violin plot displaying the difference in tumor infiltration of 22 types immune cells as calculated by CIBERSORT algorithms between the two clusters. (b–d) Boxplot showing the difference of ImmuneScore, StromalScore, and ESTIMATEScore between two clusters.

Additionally, we evaluated the TME via CIBERSORT method. As displayed in Figure 3, in TCGA cohort, the cluster 1 subtype generally had higher “B cells naive” (*p* = 0.017), while lower “NK cells resting” (*p* = 0.022), “monocytes” (*p* = 0.036), and “macrophages M0” (*p* = 0.005) when compared to cluster 2 subgroup. Besides, the cluster 1 subgroup had a higher ImmuneScore (*p* = 3.5*e* − 05), StromalScore (*p* = 3.4*e* − 05), and ESTIMATEScore (*p* = 9.8*e* − 06) than cluster 2 subgroup.

### 3.3. Necroptosis-Related Prognostic Gene Model Construction

**Figure 4:**
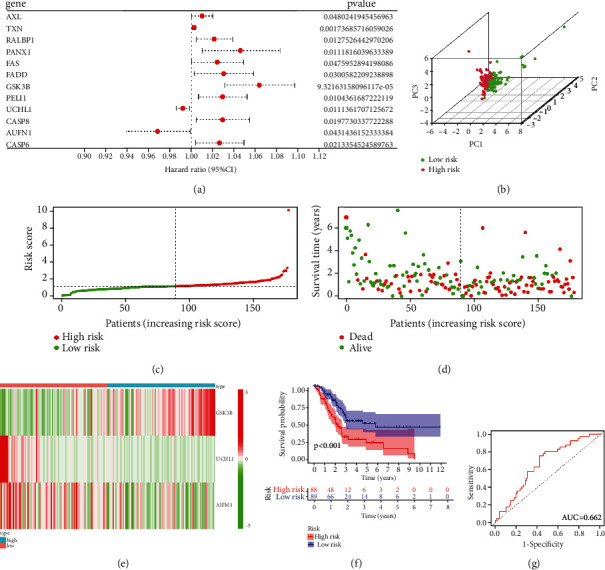
Risk model construction in TCGA cohort. (a) Univariate Cox regression analysis identified 12 necroptosis-related genes correlated with OS. (b) Principal component analysis (PCA) for PC patients according to the risk score. (c) Comparing risk score level between low- and high-risk groups. (d) Survival status for each PC patient. (e) Heatmap exhibiting the expression of the 3 identified necroptosis-related genes expression in different groups. (f) K-M survival analysis based on risk group. (g) ROC curves evaluate the sensitivity and specificity of 1-year survival prediction.

Primarily, we adopted univariate Cox regression analysis to determine the prognosis-related genes. 12 genes (AXL, TXN, RALBP1, PANX1, FAS, FADD, GSK3B, PELI1, UCHL1, CASP8, AIFM1, and CASP6) with *p* value < 0.05 were recruited for further analysis. Among these, AIFM1 and UCHL1 were correlated with decreased risk with HR < 1, while the remaining 10 genes were harmful for the prognosis of PC (Figure 4(a) and Table 1). To further narrow the potential gene numbers and build the risk model, we used multivariate Cox regression analysis. Ultimately, a 3-gene signature (GSK3B, AIFM1, and UCHL1) was constructed. The risk score = (0.05479 × the expression of GSK3B) + (−0.00712 × the expression of UCHL1) + (−0.03392 × the expression of AIFM1) (Table 2). Based on the median score in the TCGA cohort, we divided PC patients into high- and low-risk groups. The principal component analysis (PCA) suggested that the high- and low-risk groups were classified into two apparent forms of distribution, which indicated that necroptosis had significantly different role in two subgroups (Figure 4(b)). Scatter diagrams show the risk scores of each PC patient, and patients in the high-risk group had higher mortality than those in the low-risk group (Figures 4(c) and 4(d)). Besides, the heatmap plot displayed the 3 necroptosis-related genes' expression in different risk groups, as shown in Figure 4(e), GSK3B was overexpressed in high-risk group, while AIFM1 and UCHL1 were overexpressed in low-risk group. K-M survival analysis indicates that PC patients in the high-risk group had a significantly (*p* < 0.001) shorter OS than those in the low-risk group (Figure 4(f)). We used time-dependent receiver-operating characteristic (ROC) and the area under the ROC curve (AUC) to evaluate the specificity and sensitivity of the above results. And the AUC was 0.662 for 1-year, 0.666 for 3-year, and 0.802 for 5-year survival prediction (Figure 4(g), Figure S3 A, B). Furthermore, we explored the protein level of AIFM1, GSK3B, and UCHL1; immunohistochemistry (IHC) staining indicated that these three proteins were commonly overexpressed in PC tissues than in normal pancreatic samples (Figure S4).

### 3.4. Validation of the Necroptosis-Related Gene Risk Model

**Figure 5:**
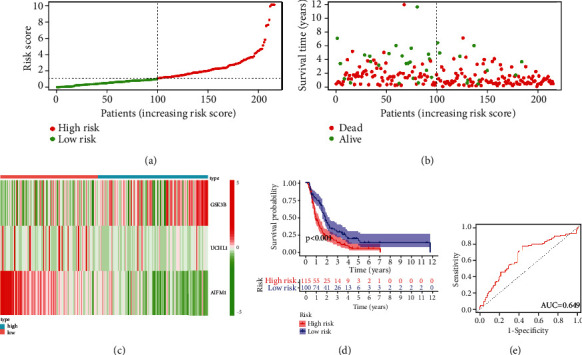
Risk model validation in ICGA cohort. (a) Risk score level between the low- and high-risk groups. (b) Survival status for each PC patient. (c) Heatmap displaying the expression of GSK3B, AIFM1, and UCHL1 in the two groups. (d) K-M survival analysis based on risk group. (e) ROC curves evaluate the 1-year survival predictive efficiency.

To further validate the prognostic efficacy of risk evaluation model, we selected the PACA-CA cohort from ICGC database. According to the median risk score of TCGA cohort, we classified the ICGA PC patients into low-risk group (100 patients) and high-risk group (115 patients) (Figure 5(a)). Similar to the TCGA cohort, the low-risk group patients had lower mortality than the high-risk group patients (Figure 5(b)). Moreover, the expression of GSK3B, AIFM1, and GSK3B was also displayed in the heatmap (Figure 5(c)). K-M survival analysis also indicates that the PC patients in the low-risk group had a significantly (*p* < 0.001) better OS than those in the high-risk group (Figure 5(d)). ROC curve also showed the risk signature had an effective sensitivity and specificity in the ICGC cohort (AUC = 0.649 for 1-year, 0.639 for 3-year, and 0.680 for 5-year survival prediction) (Figure 5(e), Figure S3 C, D).

### 3.5. Independent Prognostic Value of the Risk Signature

We performed univariate and multivariate Cox regression analysis to evaluate whether the risk model could serve as an independent prognostic factor for PC patients. As displayed in Figure S5, both univariate and multivariate Cox regression indicated that the risk score was an independent risk factor for PC patients in TCGA cohort.

### 3.6. Enrichment Analysis Based on the Risk Signature

**Figure 6:**
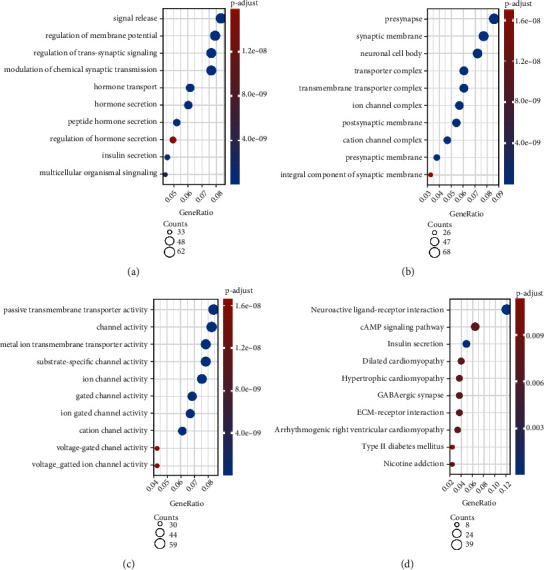
GO and KEGG enrichment analysis of DEGs based on risk groups. (a) GO: biological process (BP); (b) GO: cellular component (CC); (c) GO: molecular function (MF); (d) KEGG pathway analysis.

**Figure 7:**
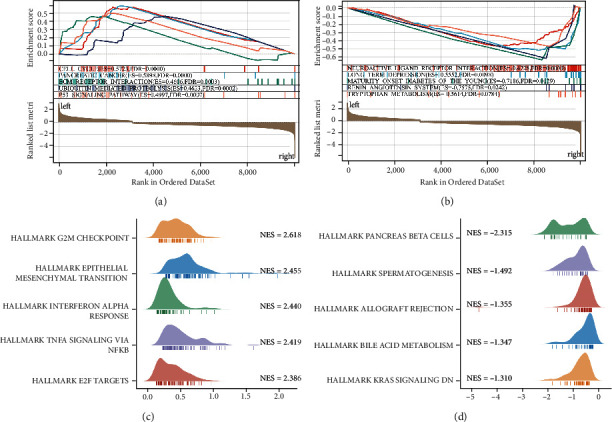
Gene Set Enrichment Analysis (GSEA). (a, b) Related signaling pathways in c2.cp.kegg.v7.4.symbols.gmt; (c, d) Related signaling pathways in h.all.v7.4.symbols.gmt.

To explore the differences in biological functions in the risk subgroups, we applied the “limma” package to screen the DEGs between these two groups with the criteria of FDR < 0.05 and absolute log2FC > 1. And the DEGs were shown in the form of a volcano map (Figure S6). GO and KEGG enrichment analysis showed that the DEGs mainly participate in “signal release,” “presynapse,” “passive transmembrane transporter activity,” and “neuroactive ligand-receptor interaction” (Figure 6). We also carried out GSEA to identify the biological pathways correlated with high- and low-risk groups in TCGA cohort. The results reveal that when “c2.cp.kegg.v7.4.symbols.gmt” was used as a reference, the high-risk group was enriched in “cell cycle,” “pancreatic cancer,” and “ECM receptor interaction,” while the low-risk group was participated in “neuroactive ligand receptor interaction,” “long term depression,” and “maturity onset diabetes of the young” (Figures 7(a) and 7(b)). When the “h.all.v7.4.symbols.gmt” was used as a reference, the high-risk group was associated with “G2M checkpoint,” “epithelial mesenchymal transition (EMT),” and “TNF*α* signaling via NF-*κ*B,” while the low-risk was enriched in “pancreas beta cells,” “spermatogenesis,” and “allograft rejection” (Figures 7(c) and 7(d)).

### 3.7. TME Analysis Based on Risk Model

**Figure 8:**
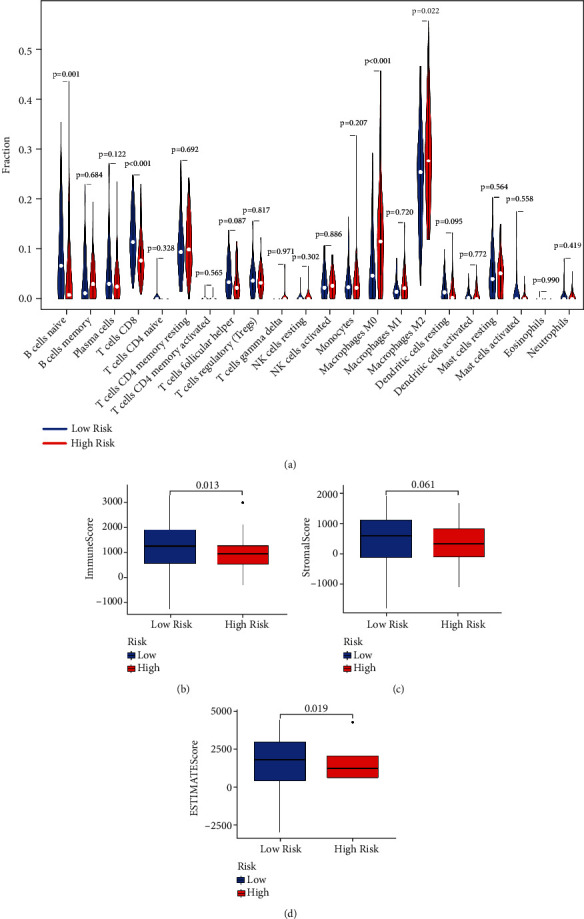
TME analysis based on two risk groups. (a) Violin plot displaying the difference in tumor infiltration of 22 types immune cells as calculated by CIBERSORT algorithms between the two groups. (b–d) Boxplot showing the difference of ImmuneScore, StromalScore, and ESTIMATEScore between two groups.

**Figure 9:**
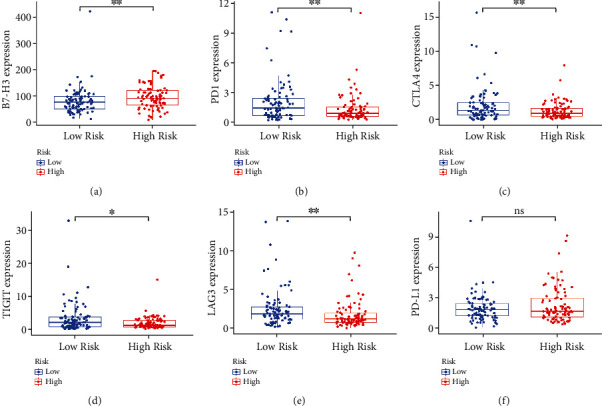
The expression difference of immune checkpoint genes between the two risk groups. (a) B7-H3 (CD276); (b) PD1 (PDCD1); (c) CTLA4; (d) TIGIT; (e) LAG3; (f) PD-L1 (CD274).

We used CIBERSORT and ESTIMATE methods to further evaluate the tumor immune cell infiltration difference between two subgroups. The results show that the high-risk group generally had higher “macrophages M0” (*p* < 0.001), “macrophages M2” (*p* = 0.022), and lower “B cells naive” (*p* = 0.001) and “CD8+ T cells” (*p* < 0.001), “when compared to the low-risk group (Figure 8(a)). In addition, the low-risk group had a higher ImmuneScore (*p* = 0.013) and ESTIMATEScore (*p* = 0.019) than the high-risk group (Figures 8(b)–8(d)). Furthermore, we explored the difference of immune checkpoint gene expression between two risk groups. As shown in Figure 9, the low-risk group was associated with a higher expression of CTLA4, LAG3, PD1(PDCD1), and TIGIT, while a lower expression of B7-H3 (CD276) than high-risk group. However, there was no difference in the expression of PD-L1 (CD274) in two subgroups.

### 3.8. Drug Susceptibility Analysis between Risk Groups

**Figure 10:**
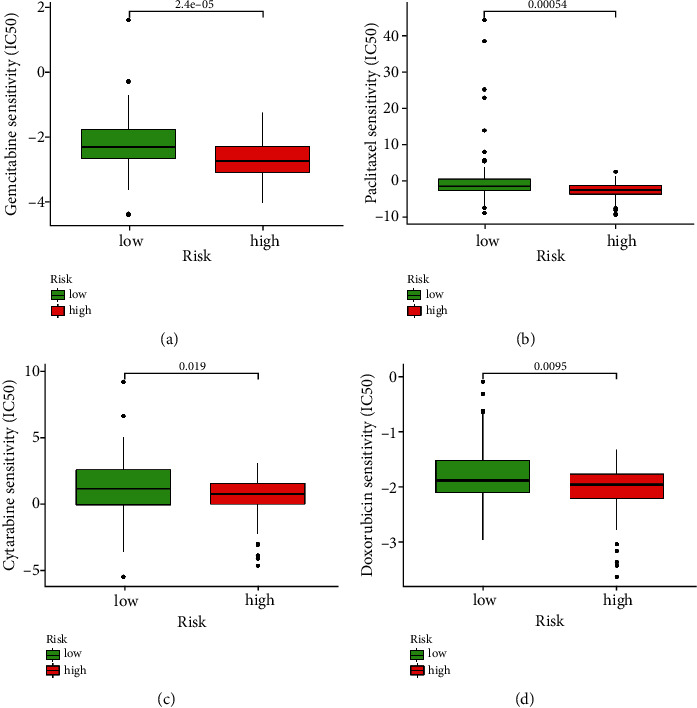
Chemotherapy sensitivity analysis between two risk groups. (a) IC50 of gemcitabine between two risk groups. (b) IC50 of paclitaxel between two risk groups. (c) IC50 of cytarabine between two risk groups. (d) IC50 of doxorubicin between two risk groups.

Noteworthy, despite the dominance of chemotherapy in the nonsurgical treatment of PC, their sensitivity is unsatisfactory to data. In order to make chemotherapy more precise, we evaluated the IC50 of various chemotherapeutic drugs between the low- and high-risk groups. The results of drug sensitivity analysis reveal that the patients in high-risk group have lower IC50s for gemcitabine, paclitaxel, cytarabine, and doxorubicin than those in the low-risk group, which indicated that the PC patients with high risk may profit from the above treatments (Figures 10(a)–10(d)).

## 4. Discussion

PC is a tumor with an extremely poor end worldwide due to its rapid progression, metastasis, ease of recurrence, and insensitivity to treatment, imposing a financial burden [13]. Additionally, majority of patients with PC are diagnosed at an advanced stage and hence lose the opportunity for surgery [2]. Although several chemotherapy options were applied for PC, such as nab-paclitaxel plus gemcitabine and mFOLFIRINOX [14, 15], the long-term survival of PC patients is still very disappointing. Moreover, the molecular mechanism of PC is still largely unknown, and novel biomarkers to predict the survival of PC patients are still unavailable. Owing to this dilemma of treatment and prognosis of PC, there is an urgency to identify an effective biomarker or model for PC.

Necroptosis, a novel defined form of necrotic cell death, was found to show a dual-effect in cancer progression and therapy target. On one hand, necroptosis could promote tumor cell necrotic and favor the antitumor immunoactivity. On the other hand, necroptosis could release immunosuppressive factors and promote tumor invasion and metastasis [16–18]. However, the interaction between necroptosis-related genes and prognosis of PC is largely unclear.

In the current research, we primarily explored the mRNA expression of 68 necroptosis-related genes in both PC and normal pancreatic tissues and identified 52 DEGs. Interestingly, all the 52 DEGs were overexpressed in PC samples when compared to normal tissues. By applying unsupervised clustering on these DEGs, we divided PC patients in the TCGA cohort into two clusters. Although the two clusters showed no significant differences in the clinicopathological features, PC patients in cluster 1 had a better survival and a higher ImmuneScore, StromalScore, and ESTIMATEScore than patients in cluster 2. To further investigate the prognostic effect of these necroptosis-related genes in PC, we employed univariate and multivariate Cox regression to construct a 3-gene (AIFM1, GSK3B, and UCHL1) risk signature for the survival prediction of PC patients. Among them, high expression of AIFM1 and UCHL1 facilitates long-term survival, while overexpression of GSK3B hampers prognosis of PC patients. Based on the median risk score, PC patients could be categorized into high- and low-risk groups, with the low-risk group patients having a longer OS than the patients of high-risk group. The PCA analysis revealed that the two groups could be effectively distinguished from each other. Similar to the previous study [19], we extracted PACA-CA dataset from the ICGC database as an external validation cohort to validate this model and obtained similar results. Moreover, univariate and multivariate Cox regression analysis suggested that the risk score could serve as an independent prognostic factor for PC. To further explore the underlying mechanisms based on the risk groups, we performed GO, KEGG, and GSEA enrichment analysis. GSEA results reveal that the high-risk group was associated with several cancer progression and metastasis-related pathways, including “cell cycle,” “pancreatic cancer,” “ECM receptor interaction,” “p53 signaling pathway,” “G2M checkpoint,” and “epithelial mesenchymal transition (EMT).” We also investigate the tumor microenvironment difference between two groups. The high-risk group had a higher M2 macrophages and lower CD8+ T cell infiltration than the low-risk patients.

Previous research revealed that GSK3B could promote DNA repair resulting in chemo- and radiotherapy resistance in glioblastoma [20]. And in PC, Namba et al. reported that inhibition of GSK3B could reverse the chemoresistance of PC cells to gemcitabine [21]. In breast cancer, the expression level of UCHL1 was negatively correlated with estrogen receptor, and inhibition of UCHL1 could enhance the sensitivity to endocrine therapy [22]. Jin et al. reported that high expression of UCHL1 was positively correlated with invasive tumor behavior and affected survival in hilar cholangiocarcinoma [23], while, in pancreatic neuroendocrine tumors, coexpression of UCHL1 and *α*-internexin predicts a better OS and disease free survival [24]. Therefore, UCHL1 may have a unique effect in different tumors. Notably, our research suggested that UCHL1 may act as an antitumor gene in PC; however, further experiments are still needed to confirm this phenomenon.

Growing evidence indicates that TME plays a significant role in the development and treatment of cancer [25, 26]. M2 macrophages could promote tumor progression and metastasis and affect therapeutic outcome of various cancer types [27–29]. Immunosurveillance plays a critical effect in the elimination of cancer cells, and CD8^+^ T cells have a vital role in antitumor. Wang et al. revealed that high CD8^+^ T cell infiltration in PC could predict a better survival [30]. Another study reported that the abundance of CD8^+^ T cells was heterogenous in PC tissues, and higher CD8^+^ T cells density was correlated with prolonged survival [31]. In our risk model, the high-risk group had a higher M2 macrophages and lower CD8^+^ T cell infiltration, which could partly explain the reason of worse prognosis of high-risk group. Similarly, the low-risk group had a higher ImmuneScore and ESTIMATEScore and indicated that the low-risk group patients had a higher abundance of immune infiltration. Besides, we assess the correlation between risk group and the expression of checkpoint gene.

Previous research revealed that chemotherapy drugs could inhibit tumor development via regulating the pathway of cell necroptosis. For instance, researchers have found that gemcitabine could induce RIPK1/RIPK3/MLKL-dependent necroptosis in cholangiocarcinoma cells [32]. Diao et al. also revealed that paclitaxel can induce phosphorylated-Casp8/RIPK1/RIPK3-dependent necroptosis in lung adenocarcinoma cells [33]. Hence, the necroptosis-related risk score model, presented in this study could serve as a valid biomarker for predicting the effect of chemotherapy in PC patients. In addition, it can provide new insights into the research of chemotherapy and necroptosis in PC.

To date, we have not seen studies of the necroptosis-related gene signature in PC, and we firstly provide insight into the role of necroptosis-related gene set in PC. We have to admit that some limitations are presented in our study. Our research is based on public databases; although we extract ICGC database as external validation, there were no our data to prove our findings. Further experiments are needed to explore the role and mechanism of the risk model in PC and to validate its clinical application.

In conclusion, our study indicated that necroptosis showed a contradictious role in PC as all the DEGs were overexpressed in PC tissues, while they showed dual role in the prognosis of PC. We successfully constructed the risk score model according to the three necroptosis-related genes; meanwhile, it could serve as an independent risk factor in the prognosis of PC patients. Based on the risk score model, the abundance level of immune cell infiltration between two groups were significant difference. Our study provides a novel gene signature for the prediction of prognosis and therapeutic markers for PC patients.

## Figures and Tables

**Table 1:** Univariate cox regression analysis of prognostic necroptosis-related genes.

Gene	HR	Lower 95% CI	Upper 95% CI	*p* value
AXL	1.010093718	1.000087323	1.020200232	0.048024195
TXN	1.002597231	1.000971072	1.004226032	0.001736857
RALBP1	1.021427284	1.004527217	1.038611675	0.012752644
PANX1	1.045866466	1.010251877	1.082736581	0.011181604
FAS	1.024210222	1.000253627	1.04874059	0.047595289
FADD	1.030214753	1.00287693	1.058297788	0.030058221
GSK3B	1.063476705	1.031149984	1.096816874	9.32E-05
PELI1	1.02926048	1.006791772	1.052230624	0.010436169
UCHL1	0.992053365	0.985960785	0.998183593	0.011136171
CASP8	1.029288755	1.00460173	1.054582438	0.019773034
AIFM1	0.968478239	0.938876822	0.999012947	0.043143615
CASP6	1.02628684	1.003862695	1.049211893	0.021335452

HR: hazard ratio; CI: confidence interval.

**Table 2:** Multivariate Cox regression analysis of prognostic necroptosis-related genes.

Gene	Coef	HR	Lower 95% CI	Upper 95% CI	*p* value
GSK3B	0.05479	1.0563	1.0237	1.0899	0.000608
UCHL1	-0.00712	0.9929	0.9864	0.9994	0.033536
AIFM1	-0.03392	0.9667	0.9362	0.9981	0.037630

Coef: coefficient; HR: hazard ratio; CI: confidence interval.

## Data Availability

The data used to support the results are available at the TCGA, GTEx, and ICGC databases.

## Abbreviations

PC: Pancreatic cancer
DEGs: Differentially expressed genes
TCGA: The Cancer Genome Atlas
ICGC: International Cancer Genome Consortium
GTEx: The Genotype-Tissue Expression
GO: Gene Ontology
KEGG: Kyoto Encyclopedia of Genes and Genomes
GSEA: Gene Set Enrichment Analysis
PPI: Protein-protein interaction
IHC: Immunohistochemistry
OS: Overall survival
PCA: Principal component analysis
ROC: Receiver-operating characteristic
EMT: Epithelial mesenchymal transition.

## Supplementary Materials

Supplementary 1 Figure S1: flow diagram of this study. Figure S2: GO and KEGG enrichment analysis of 52 necroptosis-related DEGs. Figure S3: ROC curves of risk model in survival prediction. A, B The 3- and 5-year ROC curves of risk model in TCGA cohort. C, D The 3- and 5-year ROC curves of risk model in ICGC cohort. Figure S4: characteristic IHC images of DHX32 expression in pancreatic cancer and normal pancreas tissues. A IHC staining of AIFM1 in pancreatic cancer tissues. B IHC staining of AIFM1 in normal pancreas tissues. C IHC staining of GSK3B in pancreatic cancer tissues. D IHC staining of GSK3B in normal pancreas tissues. E IHC staining of UCHL1 in pancreatic cancer tissues. F IHC staining of UCHL1 in normal pancreas tissues. Figure S5: risk score could serve as an independent prognostic factor in PC patients. Univariate (A) and multivariate (B) Cox regression analysis of risk score combining age, sex, grade, and stage. Figure S6: volcano plots show the DEGs between high- and low-risk groups.

Supplementary 2 Table S1: the necroptosis-related genes obtained from the GeneCards (http://www.genecards.org) database with the criteria of *Z*‐score > 1.
