# Supplementary figures and images for: Construction and Validation of a Necroptosis-Related Gene Signature for Predicting Prognosis and Tumor Microenvironment of Pancreatic Cancer
Source: Dis Markers. 2022 Jun 14;2022:9737587. doi: 10.1155/2022/9737587 (PMC9214653; doi:10.1155/2022/9737587)

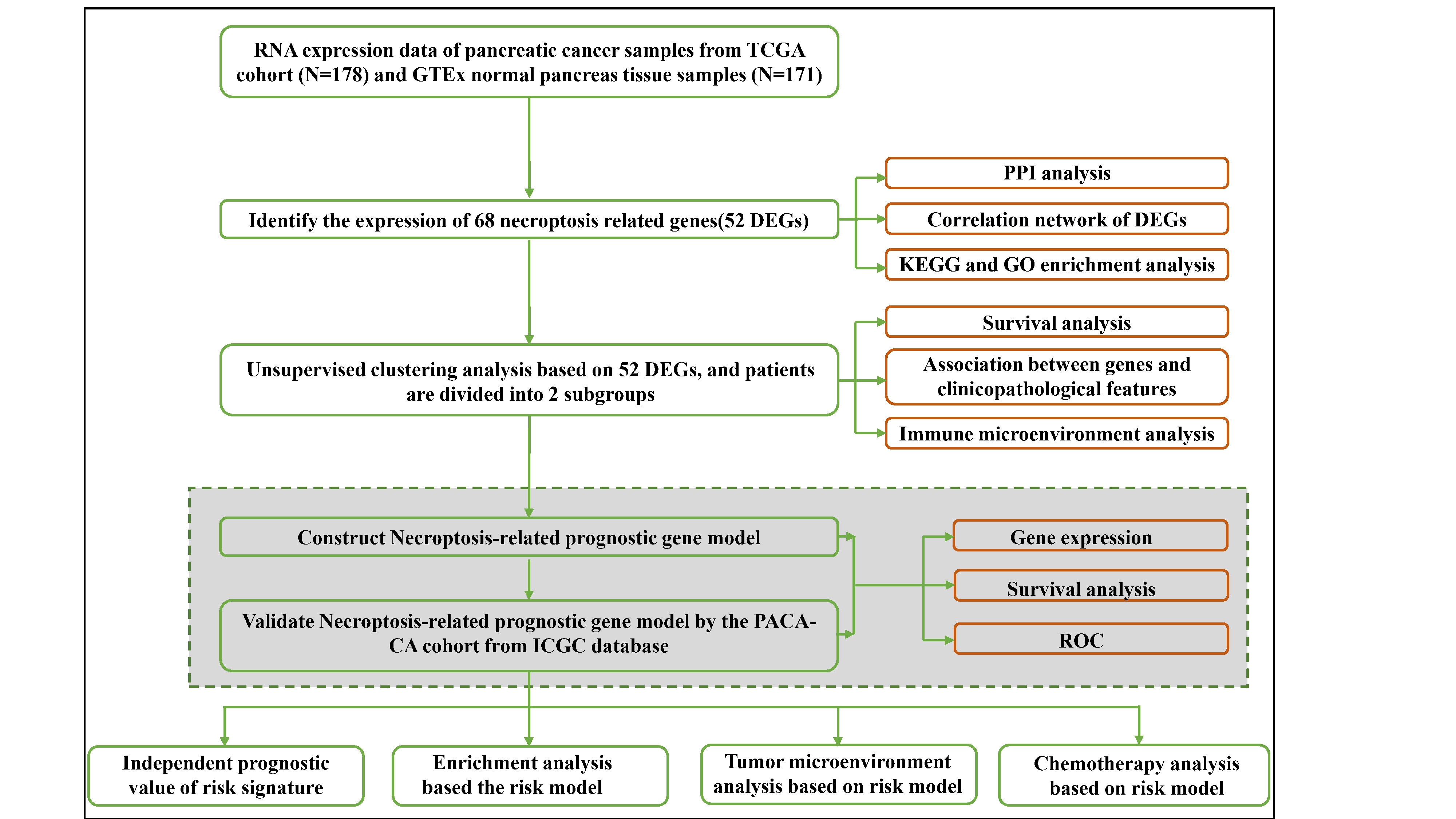

Supplement: Supplementary 1 — Figure S1: flow diagram of this study. Figure S2: GO and KEGG enrichment analysis of 52 necroptosis-related DEGs. Figure S3: ROC curves of risk model in survival prediction. A, B The 3- and 5-year ROC curves of risk model in TCGA cohort. C, D The 3- and 5-year ROC curves of risk model in ICGC cohort. Figure S4: characteristic IHC images of DHX32 expression in pancreatic cancer and normal pancreas tissues. A IHC staining of AIFM1 in pancreatic cancer tissues. B IHC staining of AIFM1 in normal pancreas tissues. C IHC staining of GSK3B in pancreatic cancer tissues. D IHC staining of GSK3B in normal pancreas tissues. E IHC staining of UCHL1 in pancreatic cancer tissues. F IHC staining of UCHL1 in normal pancreas tissues. Figure S5: risk score could serve as an independent prognostic factor in PC patients. Univariate (A) and multivariate (B) Cox regression analysis of risk score combining age, sex, grade, and stage. Figure S6: volcano plots show the DEGs between high- and low-risk groups. [file 9737587.f1.zip › FigureS1 (1).JPEG]

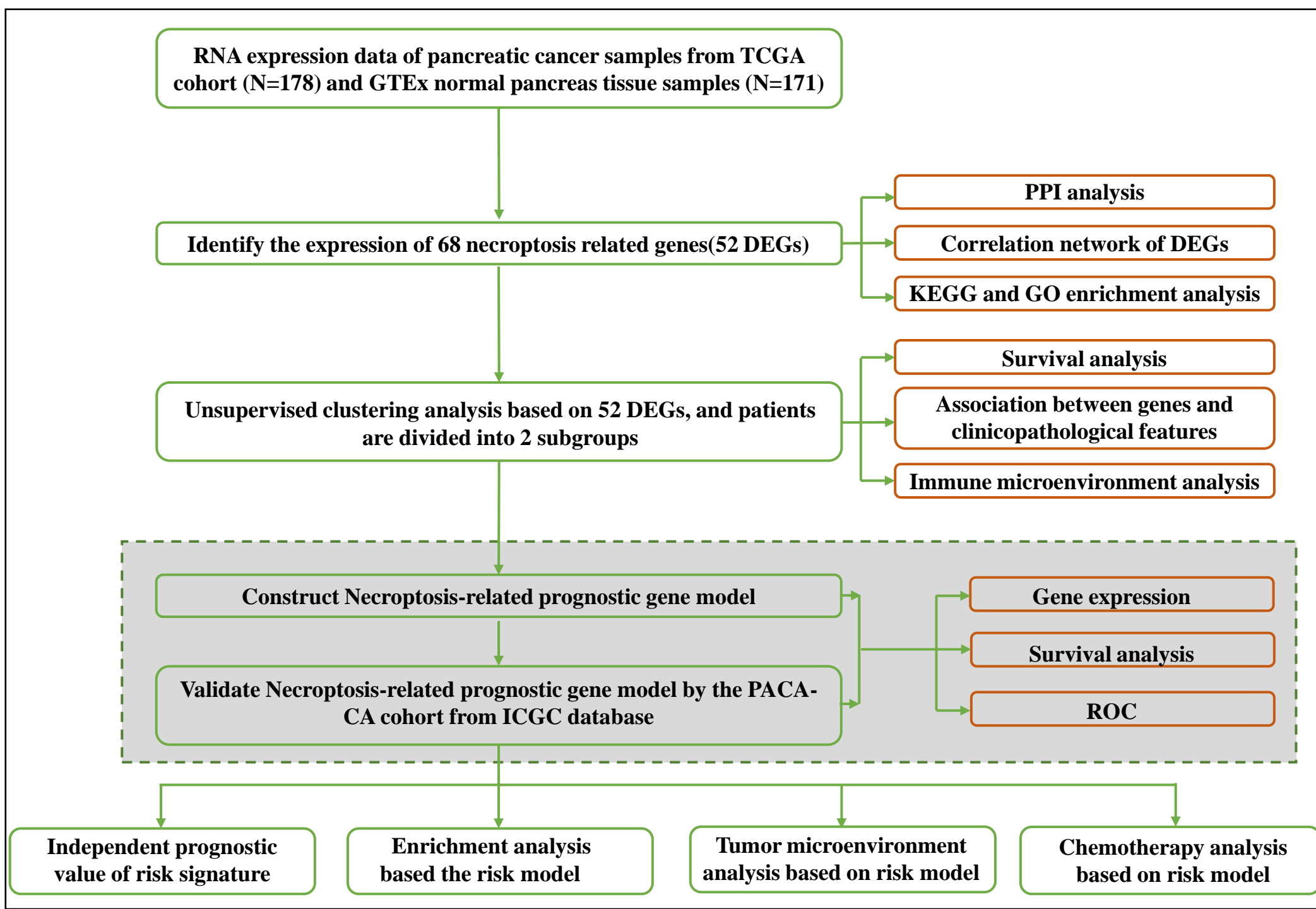

Supplement: Supplementary 1 — Figure S1: flow diagram of this study. Figure S2: GO and KEGG enrichment analysis of 52 necroptosis-related DEGs. Figure S3: ROC curves of risk model in survival prediction. A, B The 3- and 5-year ROC curves of risk model in TCGA cohort. C, D The 3- and 5-year ROC curves of risk model in ICGC cohort. Figure S4: characteristic IHC images of DHX32 expression in pancreatic cancer and normal pancreas tissues. A IHC staining of AIFM1 in pancreatic cancer tissues. B IHC staining of AIFM1 in normal pancreas tissues. C IHC staining of GSK3B in pancreatic cancer tissues. D IHC staining of GSK3B in normal pancreas tissues. E IHC staining of UCHL1 in pancreatic cancer tissues. F IHC staining of UCHL1 in normal pancreas tissues. Figure S5: risk score could serve as an independent prognostic factor in PC patients. Univariate (A) and multivariate (B) Cox regression analysis of risk score combining age, sex, grade, and stage. Figure S6: volcano plots show the DEGs between high- and low-risk groups. [file 9737587.f1.zip › FigureS1.pdf]

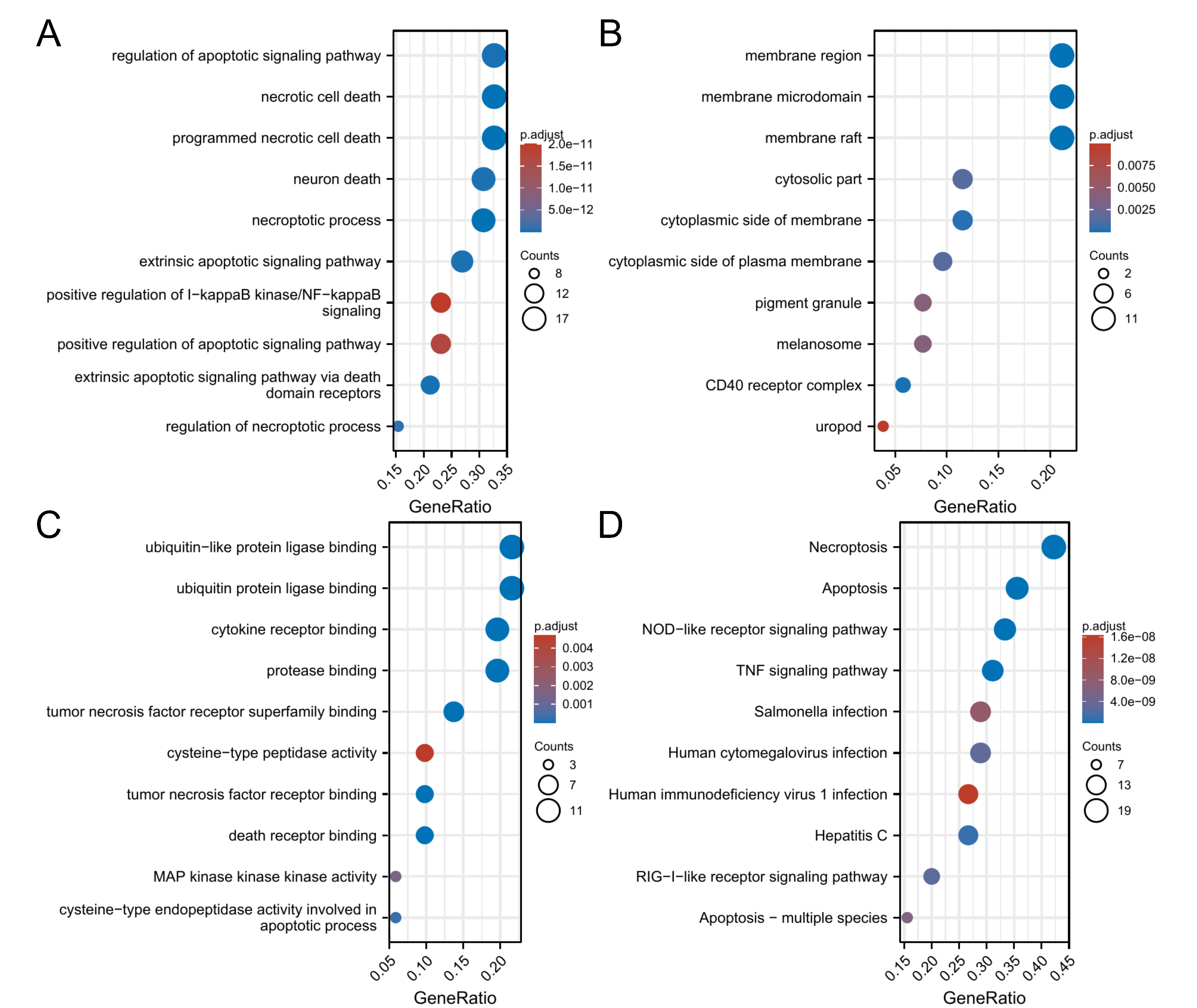

Supplement: Supplementary 1 — Figure S1: flow diagram of this study. Figure S2: GO and KEGG enrichment analysis of 52 necroptosis-related DEGs. Figure S3: ROC curves of risk model in survival prediction. A, B The 3- and 5-year ROC curves of risk model in TCGA cohort. C, D The 3- and 5-year ROC curves of risk model in ICGC cohort. Figure S4: characteristic IHC images of DHX32 expression in pancreatic cancer and normal pancreas tissues. A IHC staining of AIFM1 in pancreatic cancer tissues. B IHC staining of AIFM1 in normal pancreas tissues. C IHC staining of GSK3B in pancreatic cancer tissues. D IHC staining of GSK3B in normal pancreas tissues. E IHC staining of UCHL1 in pancreatic cancer tissues. F IHC staining of UCHL1 in normal pancreas tissues. Figure S5: risk score could serve as an independent prognostic factor in PC patients. Univariate (A) and multivariate (B) Cox regression analysis of risk score combining age, sex, grade, and stage. Figure S6: volcano plots show the DEGs between high- and low-risk groups. [file 9737587.f1.zip › FigureS2 (1).JPEG]

A

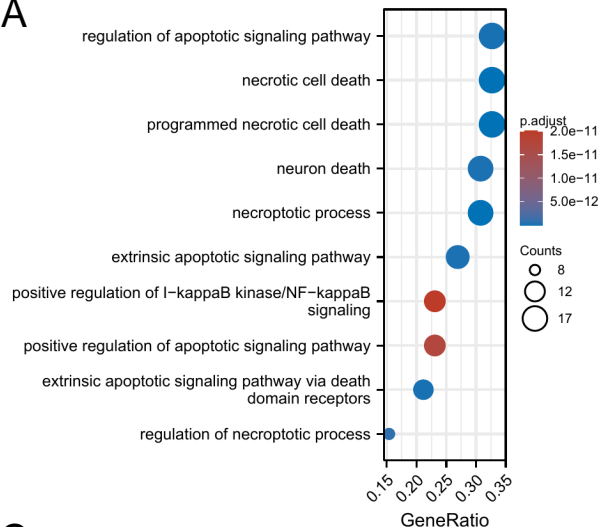

B

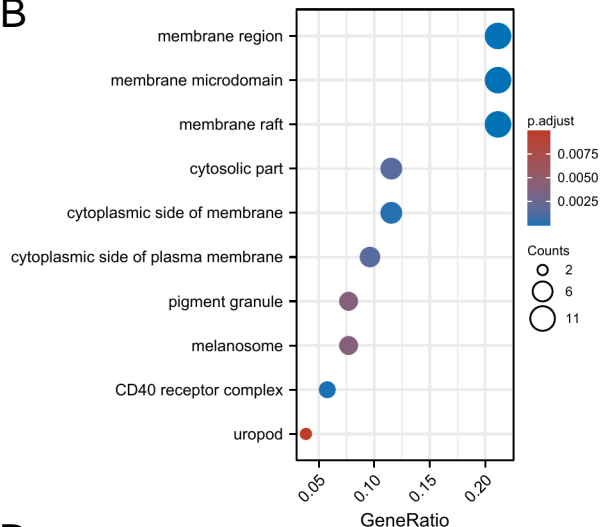

C

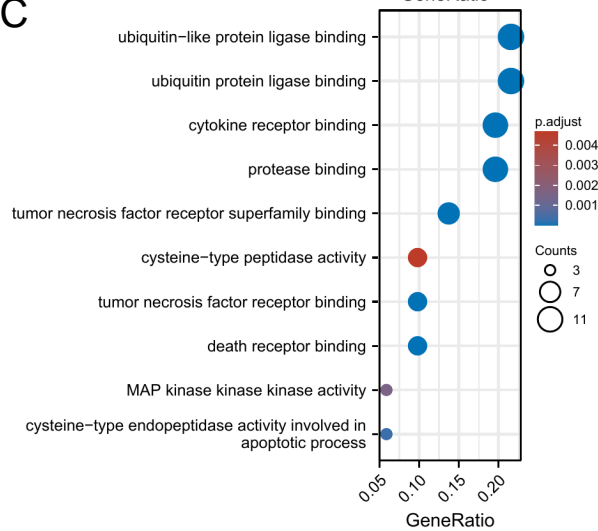

D

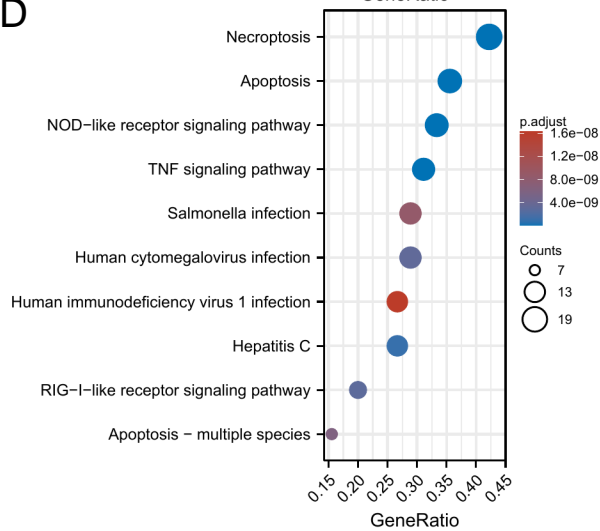

Supplement: Supplementary 1 — Figure S1: flow diagram of this study. Figure S2: GO and KEGG enrichment analysis of 52 necroptosis-related DEGs. Figure S3: ROC curves of risk model in survival prediction. A, B The 3- and 5-year ROC curves of risk model in TCGA cohort. C, D The 3- and 5-year ROC curves of risk model in ICGC cohort. Figure S4: characteristic IHC images of DHX32 expression in pancreatic cancer and normal pancreas tissues. A IHC staining of AIFM1 in pancreatic cancer tissues. B IHC staining of AIFM1 in normal pancreas tissues. C IHC staining of GSK3B in pancreatic cancer tissues. D IHC staining of GSK3B in normal pancreas tissues. E IHC staining of UCHL1 in pancreatic cancer tissues. F IHC staining of UCHL1 in normal pancreas tissues. Figure S5: risk score could serve as an independent prognostic factor in PC patients. Univariate (A) and multivariate (B) Cox regression analysis of risk score combining age, sex, grade, and stage. Figure S6: volcano plots show the DEGs between high- and low-risk groups. [file 9737587.f1.zip › FigureS2.pdf]

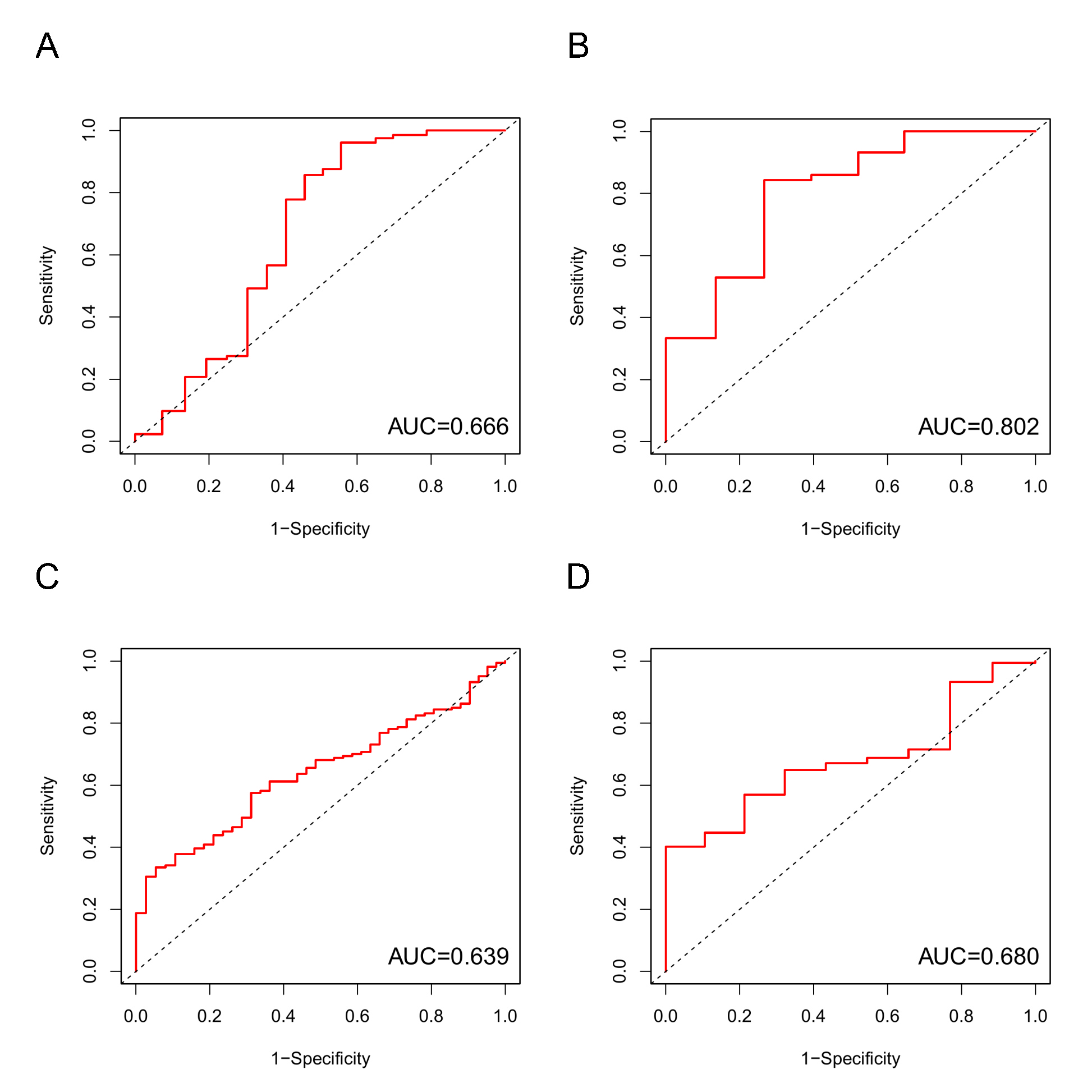

Supplement: Supplementary 1 — Figure S1: flow diagram of this study. Figure S2: GO and KEGG enrichment analysis of 52 necroptosis-related DEGs. Figure S3: ROC curves of risk model in survival prediction. A, B The 3- and 5-year ROC curves of risk model in TCGA cohort. C, D The 3- and 5-year ROC curves of risk model in ICGC cohort. Figure S4: characteristic IHC images of DHX32 expression in pancreatic cancer and normal pancreas tissues. A IHC staining of AIFM1 in pancreatic cancer tissues. B IHC staining of AIFM1 in normal pancreas tissues. C IHC staining of GSK3B in pancreatic cancer tissues. D IHC staining of GSK3B in normal pancreas tissues. E IHC staining of UCHL1 in pancreatic cancer tissues. F IHC staining of UCHL1 in normal pancreas tissues. Figure S5: risk score could serve as an independent prognostic factor in PC patients. Univariate (A) and multivariate (B) Cox regression analysis of risk score combining age, sex, grade, and stage. Figure S6: volcano plots show the DEGs between high- and low-risk groups. [file 9737587.f1.zip › FigureS3 (1).JPEG]

A

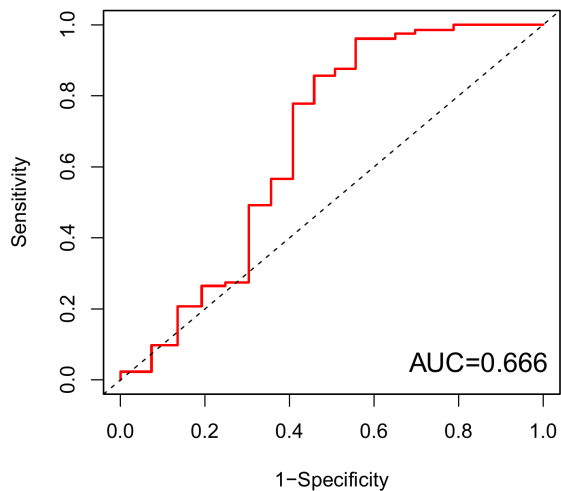

B

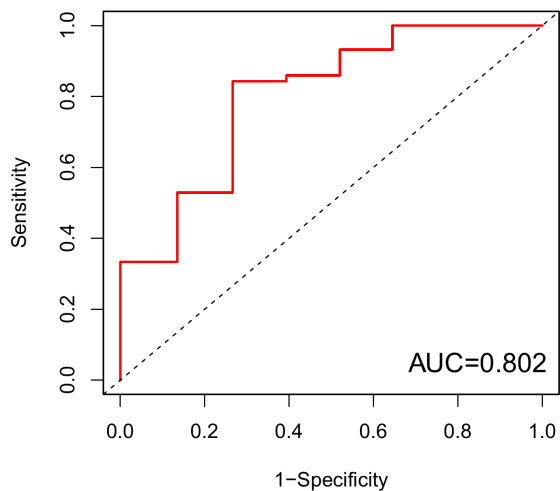

C

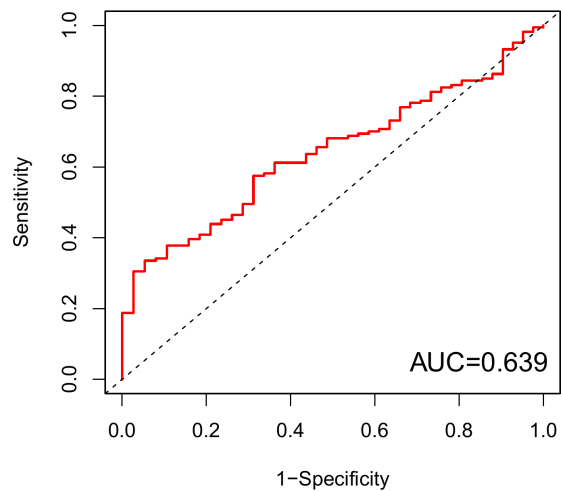

D

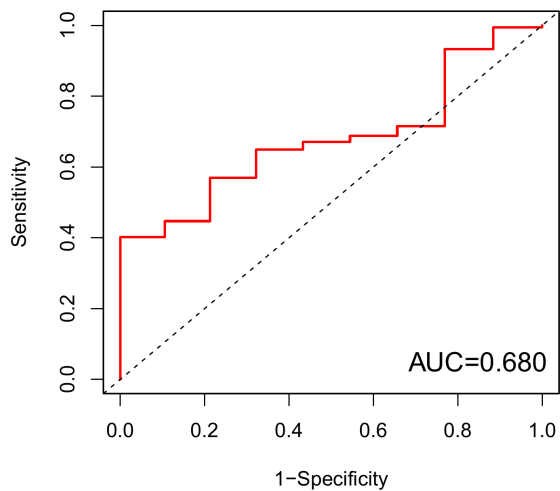

Supplement: Supplementary 1 — Figure S1: flow diagram of this study. Figure S2: GO and KEGG enrichment analysis of 52 necroptosis-related DEGs. Figure S3: ROC curves of risk model in survival prediction. A, B The 3- and 5-year ROC curves of risk model in TCGA cohort. C, D The 3- and 5-year ROC curves of risk model in ICGC cohort. Figure S4: characteristic IHC images of DHX32 expression in pancreatic cancer and normal pancreas tissues. A IHC staining of AIFM1 in pancreatic cancer tissues. B IHC staining of AIFM1 in normal pancreas tissues. C IHC staining of GSK3B in pancreatic cancer tissues. D IHC staining of GSK3B in normal pancreas tissues. E IHC staining of UCHL1 in pancreatic cancer tissues. F IHC staining of UCHL1 in normal pancreas tissues. Figure S5: risk score could serve as an independent prognostic factor in PC patients. Univariate (A) and multivariate (B) Cox regression analysis of risk score combining age, sex, grade, and stage. Figure S6: volcano plots show the DEGs between high- and low-risk groups. [file 9737587.f1.zip › FigureS3.pdf]

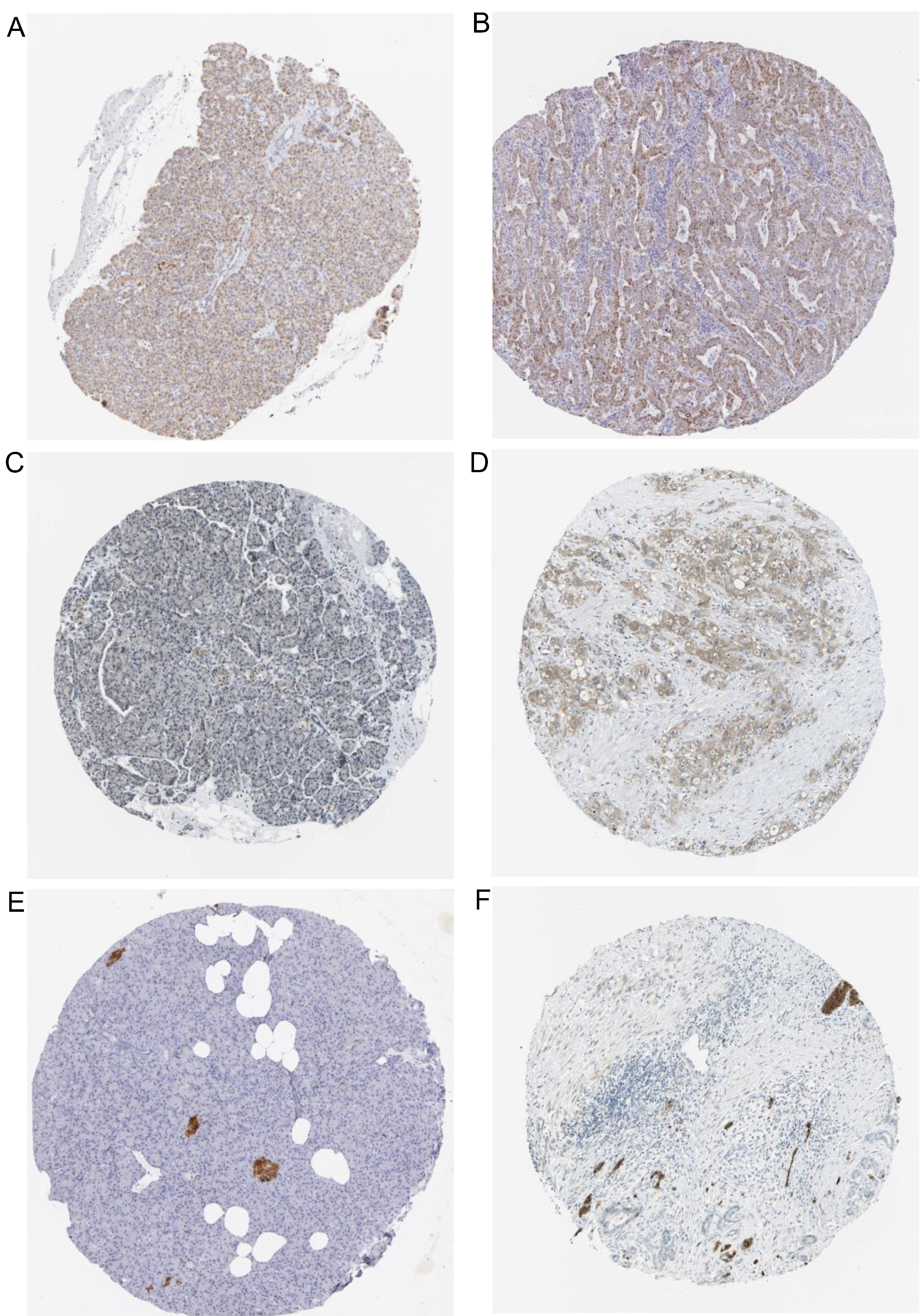

Supplement: Supplementary 1 — Figure S1: flow diagram of this study. Figure S2: GO and KEGG enrichment analysis of 52 necroptosis-related DEGs. Figure S3: ROC curves of risk model in survival prediction. A, B The 3- and 5-year ROC curves of risk model in TCGA cohort. C, D The 3- and 5-year ROC curves of risk model in ICGC cohort. Figure S4: characteristic IHC images of DHX32 expression in pancreatic cancer and normal pancreas tissues. A IHC staining of AIFM1 in pancreatic cancer tissues. B IHC staining of AIFM1 in normal pancreas tissues. C IHC staining of GSK3B in pancreatic cancer tissues. D IHC staining of GSK3B in normal pancreas tissues. E IHC staining of UCHL1 in pancreatic cancer tissues. F IHC staining of UCHL1 in normal pancreas tissues. Figure S5: risk score could serve as an independent prognostic factor in PC patients. Univariate (A) and multivariate (B) Cox regression analysis of risk score combining age, sex, grade, and stage. Figure S6: volcano plots show the DEGs between high- and low-risk groups. [file 9737587.f1.zip › FigureS4 (1).JPEG]

A

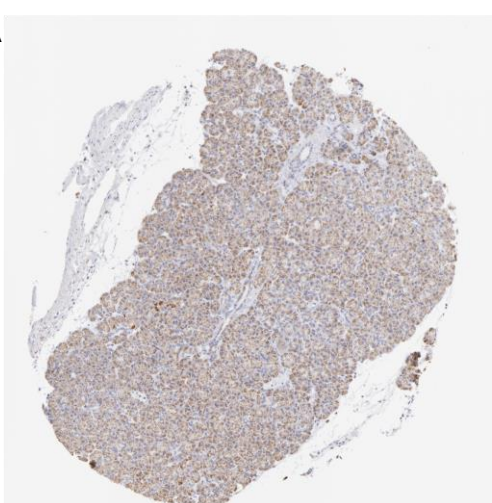

B

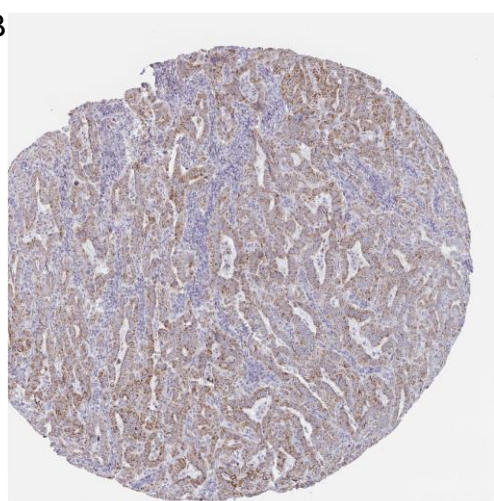

C

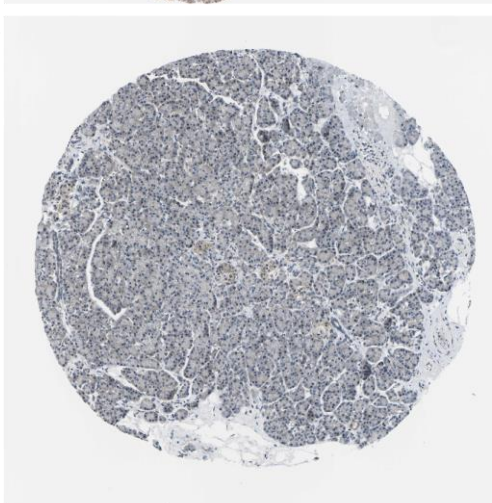

D

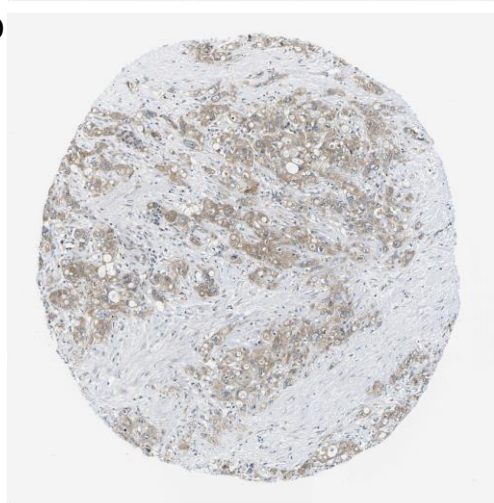

E

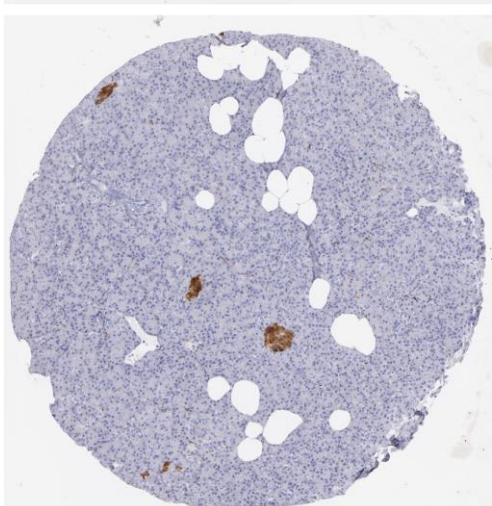

F

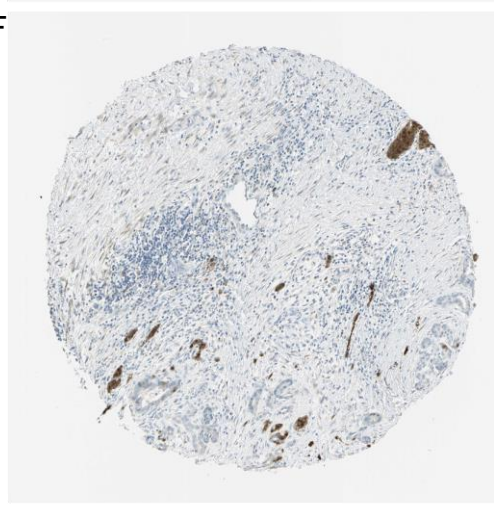

Supplement: Supplementary 1 — Figure S1: flow diagram of this study. Figure S2: GO and KEGG enrichment analysis of 52 necroptosis-related DEGs. Figure S3: ROC curves of risk model in survival prediction. A, B The 3- and 5-year ROC curves of risk model in TCGA cohort. C, D The 3- and 5-year ROC curves of risk model in ICGC cohort. Figure S4: characteristic IHC images of DHX32 expression in pancreatic cancer and normal pancreas tissues. A IHC staining of AIFM1 in pancreatic cancer tissues. B IHC staining of AIFM1 in normal pancreas tissues. C IHC staining of GSK3B in pancreatic cancer tissues. D IHC staining of GSK3B in normal pancreas tissues. E IHC staining of UCHL1 in pancreatic cancer tissues. F IHC staining of UCHL1 in normal pancreas tissues. Figure S5: risk score could serve as an independent prognostic factor in PC patients. Univariate (A) and multivariate (B) Cox regression analysis of risk score combining age, sex, grade, and stage. Figure S6: volcano plots show the DEGs between high- and low-risk groups. [file 9737587.f1.zip › FigureS4.pdf]

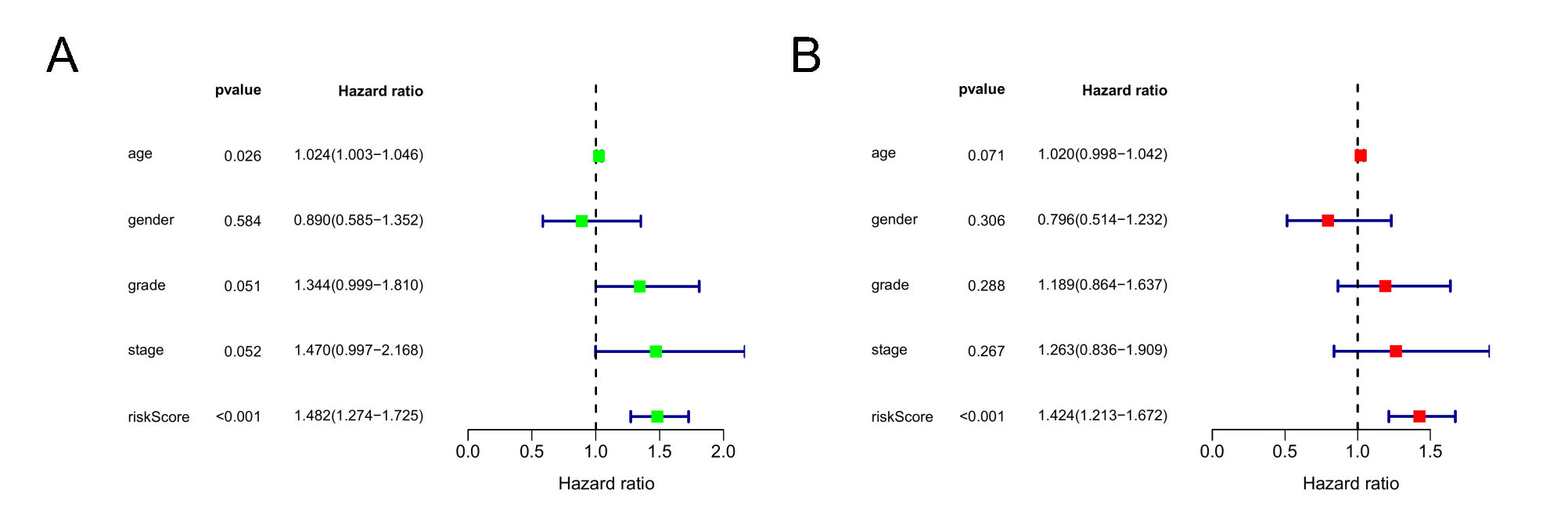

Supplement: Supplementary 1 — Figure S1: flow diagram of this study. Figure S2: GO and KEGG enrichment analysis of 52 necroptosis-related DEGs. Figure S3: ROC curves of risk model in survival prediction. A, B The 3- and 5-year ROC curves of risk model in TCGA cohort. C, D The 3- and 5-year ROC curves of risk model in ICGC cohort. Figure S4: characteristic IHC images of DHX32 expression in pancreatic cancer and normal pancreas tissues. A IHC staining of AIFM1 in pancreatic cancer tissues. B IHC staining of AIFM1 in normal pancreas tissues. C IHC staining of GSK3B in pancreatic cancer tissues. D IHC staining of GSK3B in normal pancreas tissues. E IHC staining of UCHL1 in pancreatic cancer tissues. F IHC staining of UCHL1 in normal pancreas tissues. Figure S5: risk score could serve as an independent prognostic factor in PC patients. Univariate (A) and multivariate (B) Cox regression analysis of risk score combining age, sex, grade, and stage. Figure S6: volcano plots show the DEGs between high- and low-risk groups. [file 9737587.f1.zip › FigureS5.JPEG]

A

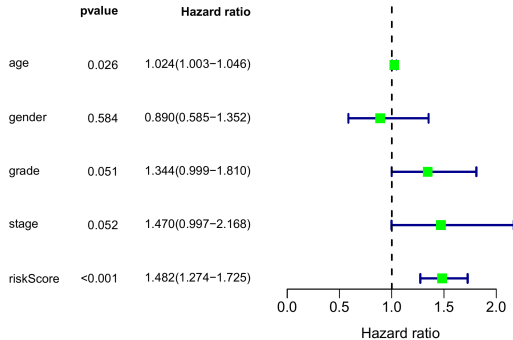

B

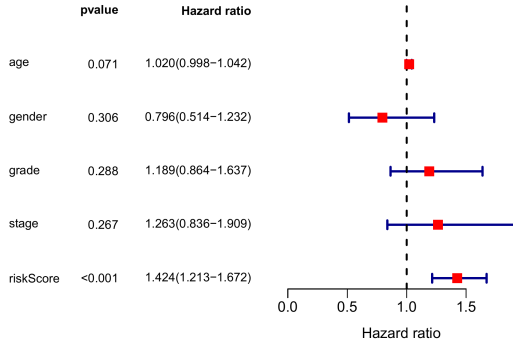

Supplement: Supplementary 1 — Figure S1: flow diagram of this study. Figure S2: GO and KEGG enrichment analysis of 52 necroptosis-related DEGs. Figure S3: ROC curves of risk model in survival prediction. A, B The 3- and 5-year ROC curves of risk model in TCGA cohort. C, D The 3- and 5-year ROC curves of risk model in ICGC cohort. Figure S4: characteristic IHC images of DHX32 expression in pancreatic cancer and normal pancreas tissues. A IHC staining of AIFM1 in pancreatic cancer tissues. B IHC staining of AIFM1 in normal pancreas tissues. C IHC staining of GSK3B in pancreatic cancer tissues. D IHC staining of GSK3B in normal pancreas tissues. E IHC staining of UCHL1 in pancreatic cancer tissues. F IHC staining of UCHL1 in normal pancreas tissues. Figure S5: risk score could serve as an independent prognostic factor in PC patients. Univariate (A) and multivariate (B) Cox regression analysis of risk score combining age, sex, grade, and stage. Figure S6: volcano plots show the DEGs between high- and low-risk groups. [file 9737587.f1.zip › FigureS5.pdf]

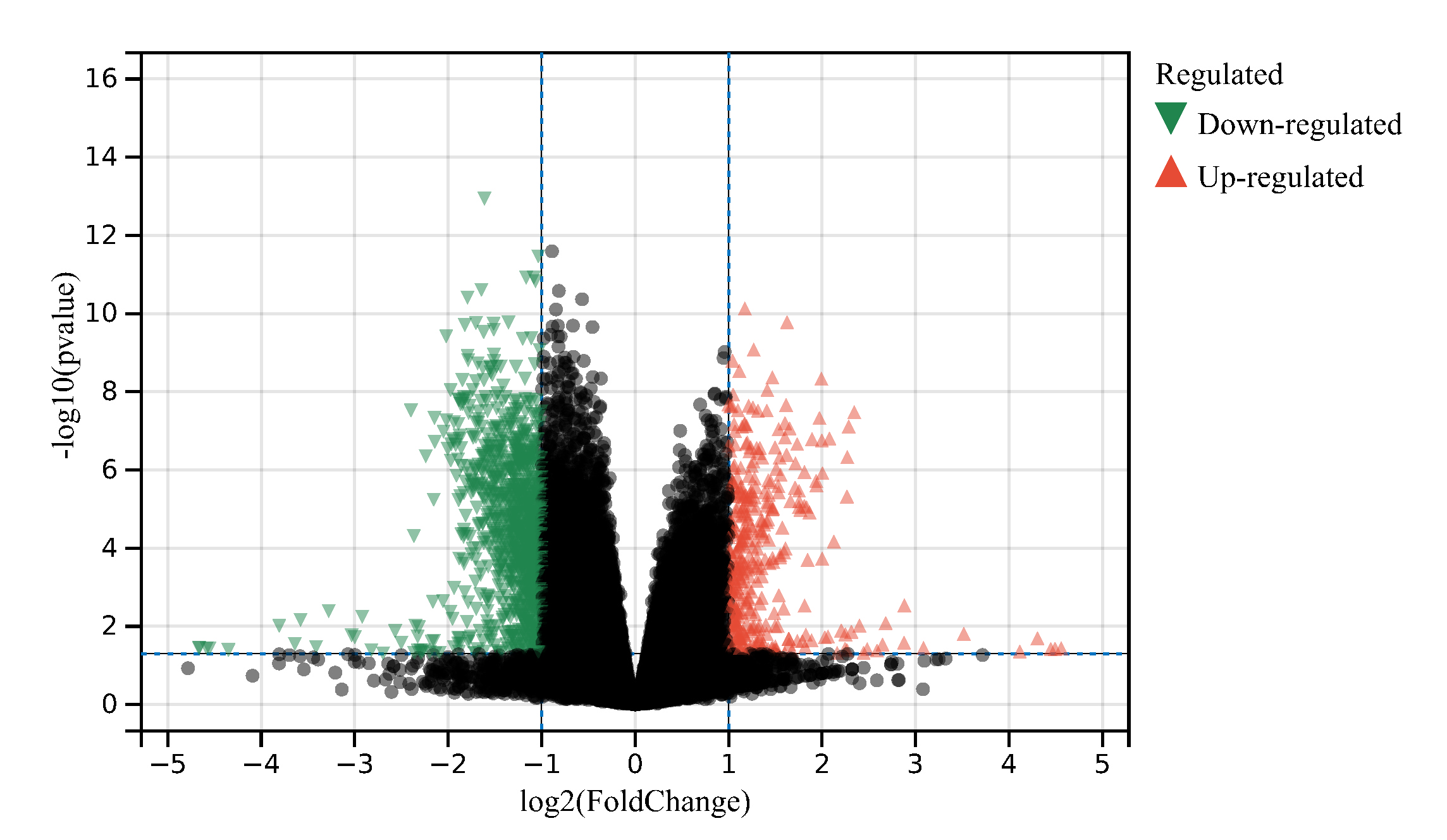

Supplement: Supplementary 1 — Figure S1: flow diagram of this study. Figure S2: GO and KEGG enrichment analysis of 52 necroptosis-related DEGs. Figure S3: ROC curves of risk model in survival prediction. A, B The 3- and 5-year ROC curves of risk model in TCGA cohort. C, D The 3- and 5-year ROC curves of risk model in ICGC cohort. Figure S4: characteristic IHC images of DHX32 expression in pancreatic cancer and normal pancreas tissues. A IHC staining of AIFM1 in pancreatic cancer tissues. B IHC staining of AIFM1 in normal pancreas tissues. C IHC staining of GSK3B in pancreatic cancer tissues. D IHC staining of GSK3B in normal pancreas tissues. E IHC staining of UCHL1 in pancreatic cancer tissues. F IHC staining of UCHL1 in normal pancreas tissues. Figure S5: risk score could serve as an independent prognostic factor in PC patients. Univariate (A) and multivariate (B) Cox regression analysis of risk score combining age, sex, grade, and stage. Figure S6: volcano plots show the DEGs between high- and low-risk groups. [file 9737587.f1.zip › FigureS6.JPEG]

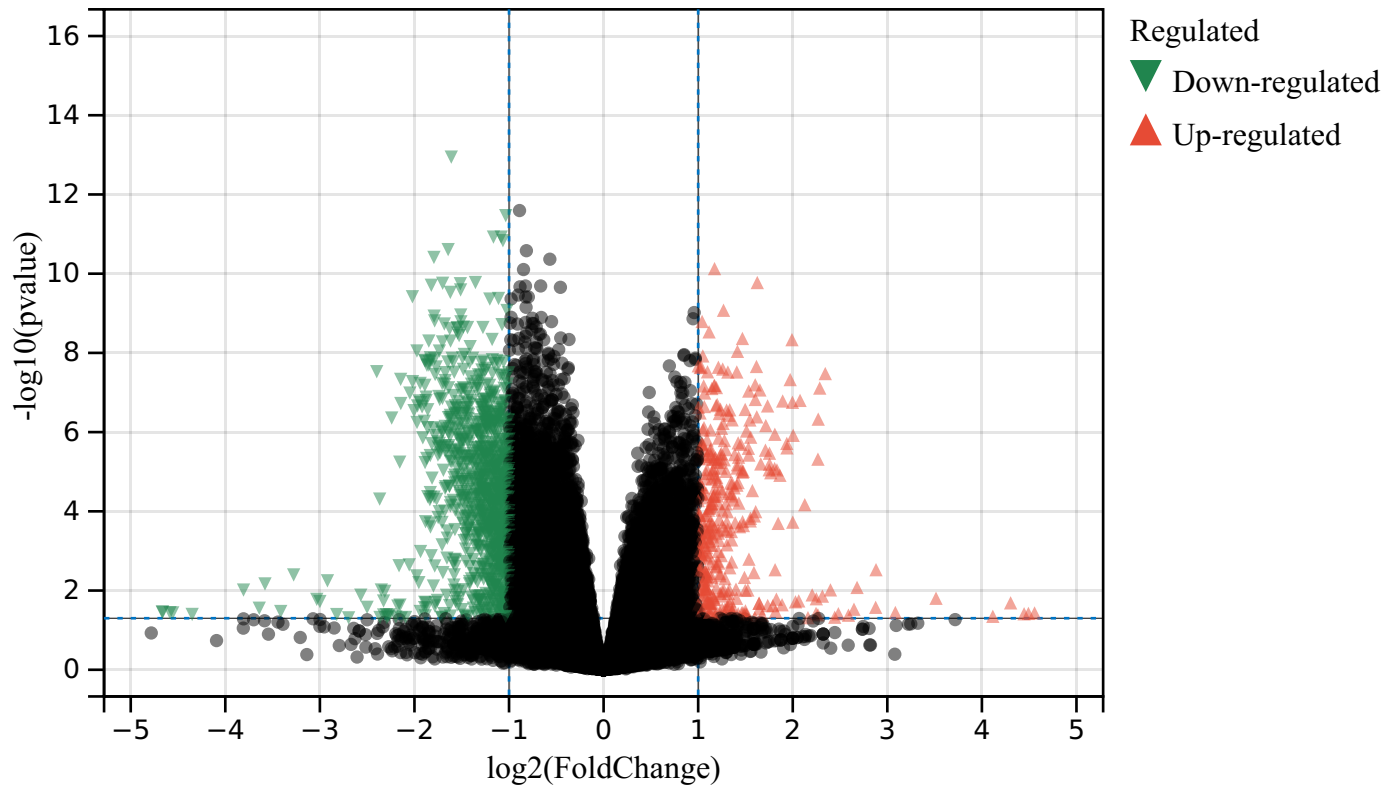

Supplement: Supplementary 1 — Figure S1: flow diagram of this study. Figure S2: GO and KEGG enrichment analysis of 52 necroptosis-related DEGs. Figure S3: ROC curves of risk model in survival prediction. A, B The 3- and 5-year ROC curves of risk model in TCGA cohort. C, D The 3- and 5-year ROC curves of risk model in ICGC cohort. Figure S4: characteristic IHC images of DHX32 expression in pancreatic cancer and normal pancreas tissues. A IHC staining of AIFM1 in pancreatic cancer tissues. B IHC staining of AIFM1 in normal pancreas tissues. C IHC staining of GSK3B in pancreatic cancer tissues. D IHC staining of GSK3B in normal pancreas tissues. E IHC staining of UCHL1 in pancreatic cancer tissues. F IHC staining of UCHL1 in normal pancreas tissues. Figure S5: risk score could serve as an independent prognostic factor in PC patients. Univariate (A) and multivariate (B) Cox regression analysis of risk score combining age, sex, grade, and stage. Figure S6: volcano plots show the DEGs between high- and low-risk groups. [file 9737587.f1.zip › FigureS6.pdf]
