# Supplementary material for: Construction and Validation of a Necroptosis-Related Gene Signature for Predicting Prognosis and Tumor Microenvironment of Pancreatic Cancer
Source: Dis Markers. 2022 Jun 14;2022:9737587. doi: 10.1155/2022/9737587 (PMC9214653; doi:10.1155/2022/9737587)
Supplement: Supplementary 2 — Table S1: the necroptosis-related genes obtained from the GeneCards (http://www.genecards.org) database with the criteria of Z‐score > 1. [file 9737587.f2.docx]

The necroptosis-related genes obtained from GeneCards database

| CHMP4B |
| --- |
| RALBP1 |
| TXN |
| SERTAD1 |
| GSK3B |
| STING1 |
| FADD |
| ITPK1 |
| TRAF2 |
| FKBP1A |
| DIABLO |
| XIAP |
| PANX1 |
| AXL |
| PTGES3 |
| TNFRSF1A |
| SLC39A7 |
| HTRA2 |
| TIMM50 |
| AIFM1 |
| FLOT1 |
| FLOT2 |
| PELI1 |
| CXCL5 |
| FAS |
| ZBP1 |
| CYLD |
| GNLY |
| TP53 |
| CFLAR |
| PITPNA |
| CASP8 |
| MAP3K7 |
| DNM1L |
| SPATA2 |
| MAPK14 |
| HSPA5 |
| RIPK1 |
| IPMK |
| RB1 |
| BIRC2 |
| PDCD6IP |
| SIRT2 |
| GSDMD |
| CASP6 |
| PARP1 |
| MLKL |
| RIPK3 |
| FASN |
| TNF |
| UCHL1 |
| FASLG |
| TP53I3 |
| BCL2 |
| PRKAA1 |
| EZH2 |
| BRD4 |
| PRKN |
| USP22 |
| TRPM7 |
| SIRT3 |
| TYRO3 |
| MYC |
| DAPK1 |
| PRKAA2 |
| MERTK |
| SLC25A37 |
| PGLYRP1 |
